# Prevalence and incidence of moderate and severe mental illness in the second postpartum year in England (1995–2020): a national retrospective cohort study using primary care data

**DOI:** 10.1016/j.lanepe.2025.101312

**Published:** 2025-05-09

**Authors:** Ellie Jones, Laura Quinn, Jamie-Rae Tanner, Jelena Jankovic, Giles Berrisford, Christine MacArthur, Beck Taylor

**Affiliations:** a Department of Applied Health Sciences, School of Applied Health Sciences, College of Medicine and Health, University of Birmingham, Edgbaston, Birmingham, B15 2TT, United Kingdom; b Warwickshire County Council, Shire Hall, Market Place, Warwick, CV344RL, United Kingdom; c Birmingham and Solihull Mental Health Foundation Trust, Perinatal Mental Health Service, The Barberry, 25 Vincent Drive, Birmingham, B15 3RB, United Kingdom; d Maudsley Health, 201-205 Al Montazah Tower, Khalidiyah, Abu Dhabi, 41763, United Arab Emirates; e Warwick Medical School, University of Warwick, Coventry, CV4 7AL, United Kingdom

**Keywords:** Perinatal mental health, Perinatal mental illness, Psychiatry

## Abstract

**Background:**

Perinatal mental illness affects around 20% of women in pregnancy and the first postpartum year with little evidence regarding persistence and incidence in the second year. This study aimed to describe prevalence and incidence of moderate and severe mental illness in the second postpartum year to estimate the proportion of women who could benefit from extension of England's specialist perinatal mental health services to two years.

**Methods:**

A retrospective cohort study using United Kingdom primary care Clinical Practice Research Datalink GOLD. All women registered with a General Practitioner with third trimester, delivery code or postpartum medical record 1995–2020 were included. Secondary objectives were to investigate mental illness type and associated factors.

**Findings:**

2,132,754 pregnancies from 1,361,497 women were included. Prevalence of mental illness likely to need specialist PMH services in second postpartum year increased significantly from 3.1% (n = 2643/85,756) in 1995 to 7.4% (n = 2473/34,098) in 2018. Incident cases increased from 1.9% (n = 1630/85,756) in 1995 to 3.8% (n = 1285/34,098) in 2018 representing 56.6% (n = 69,926/123,510) of all cases in the second year. Adjusted analysis showed odds of mental illness in second year were higher: for women in most ages vs 30–34 yrs; for each additional pregnancy (OR: 1.16, 95% CI: 1.13, 1.19 two vs one); for preterm births (OR: 1.21, 95% CI: 1.15, 1.27), near term (OR: 1.21, 95% CI: 1.17, 1.25) or post-term (OR: 1.07, 95% CI: 1.04, 1.09) vs term; with history of mental illness (OR: 2.46, 95% CI: 2.41, 2.52), smoking (OR: 1.37, 95% CI: 1.35, 1.39), substance use disorder (OR: 1.54, 95% CI: 1.48, 1.60), and for each year vs 1995. Separate analysis using a subset of data showed odds of mental illness were higher for women in all quintiles vs least deprived and for women of white ethnicity vs all other ethnicities. Although severity could not be accurately measured, most recorded illnesses would require specialist perinatal mental health input.

**Interpretation:**

Extension of specialist perinatal mental health services to two years postpartum is justified.

**Funding:**

National Institute for Health and Care Research Applied Research Collaboration West Midlands (NIHR200165).

**Research in context**

**Evidence before this study**

There is little evidence regarding prevalence and incidence of mental illness beyond the first postpartum year. In 2019, the NHS in England secured Government resource to ensure that by 2023/24, an additional 24,000 women with moderate to severe perinatal mental illness would have access to specialist perinatal mental health (PMH) care from preconception to 24 months after birth, extended from 12 months. MEDLINE, EMBASE, and PsycINFO were systematically searched from inception to 14/11/2024 for evidence of the prevalence and incidence of postpartum mental illness beyond the first year after giving birth using the following terms ([“mental illness”, “mental health problem” “mental disorder” “mental stress” “schizophrenia” “serious mental illness” “depression” “anxiety” “personality disorder” “bipolar disorder” “obessive compulsive disorder” “post-traumatic stress disorder”] AND [“second postpartum year”, “second postnatal year”] AND [“prevalence”, “incidence”]. No studies estimating prevalence or incidence of mental illness in the second year postpartum were identified, therefore it was not known how many women would be eligible for the service, or whether the proposed additional resources from NHS England would meet demand. One UK Cohort study investigated prevalence of maternal mental illness among children and adolescents but did not specifically explore prevalence and incidence of mental illness in the second postpartum year. Another cohort study in Scotland and found elevated risk of admission to psychiatric hospital for women until the end of the second postpartum year.

**Added Value of this study**

This study uses a nationally representative database of over 2.1 million pregnancies in 1.3 million women to determine the prevalence and incidence of mental illness in the second postpartum year. It showed that the prevalence of women with a recorded mental illness and likely to be eligible for NHS PMH care in the second year was 5.9%, with 3.3% cases being new onset. The comparable prevalence for the first year from this dataset was 7.5% with 6.6% being new onset.

**Implications of all the available evidence**

The findings from the study indicate considerable ongoing need for specialist PMH services in the second postpartum year, supporting the extension of UK perinatal services up to two years.

## Introduction

Mental illness in pregnancy and the first year after birth affects up to 20% of women globally,^1^ and in the UK costs the National Health Service (NHS) around £1.2 billion per annual cohort of births.^2^ Perinatal mental illness (PMI) varies in severity and depression and anxiety are the most common type.^3^ Risk factors for PMI include previous history of mental illness, deprivation, life stress and poor social relationships.^4^, ^5^, ^6^ Without treatment, PMI can lead to poor psychological and employment outcomes for women, and a range of adverse child outcomes which can persist into late adolescence.^3^^,^^7^ Although rare, psychiatric-related deaths are one of the leading causes of maternal death in the UK.^8^

There is overwhelming evidence that specialist (psychiatric and psychological) perinatal mental health (PMH) services are necessary to improve outcomes for women and babies^3^ and economic modelling suggests cost effectiveness.^2^ However, what is less clear is for how long women should be able to access specialist PMH services or where task-sharing with non-specialist mental healthcare providers such as midwives, nurses and GPs^9^^,^^10^ or psychological treatment in non-perinatal mental health provision such as the NHS's Talking Therapies is sustainable and sufficient.^11^ Traditionally, PMI has been defined as mental illness during pregnancy and up to a year postpartum,^3^ however, it has been argued that the 12-month cut-off may be somewhat arbitrary. There is increasing evidence that the first 1001 days of life are the most critical where consistency of healthcare provision is beneficial, particularly for new mothers with poor mental health struggling to bond with their babies.^12^ A Scottish cohort study found elevated risk of admission to a psychiatric hospital up to two years after giving birth compared to pre-pregnancy period.^13^ Furthermore, psychiatric-related maternal deaths occur more frequently in the latter half of the first postpartum year,^8^ with suicide risk peaking towards the end of the first postpartum year^14^ which indicates that PMI often does not resolve at 12 months postpartum.

In 2019, NHS England secured Government resource to ensure that by 2023/24 an additional 24,000 women per year with moderate to severe PMI would have access to specialist PMH care from preconception to 24 months after birth, including availability of specialist PMH community care, improved access to evidence-based psychological, and mental health checks for partners of women accessing PMH services. ^15^ Previously, only women with severe mental illness would have access to PMH services and would be transferred to adult psychiatric or primary care General Practitioner (GP) services at 12 months postpartum. It is not known whether the additional NHS funding for the newly proposed two year postpartum ‘cut-off’ for access to PMH services for women with severe and moderate PMI is appropriate to the scale of need. Whilst recent studies have examined prevalence of maternal mental illness in pregnancy,^16^ and mental illness in the mothers of children and adolescents,^6^ there is little information that explores prevalence and incidence of mental illness specifically in the second postpartum year, nor in any subsequent years. This study aimed to describe the prevalence and incidence of mental illness in women who consulted their GP in the second postpartum year and factors associated with this.

## Methods

### Study design

A retrospective cohort study design using electronic primary care data was used.

### Data sources

Data for this study were obtained from the UK Clinical Practice Research Datalink (CPRD) GOLD, a primary care database of computerised registered patient records from GP practices in the UK covering approximately 8% of the UK population. CPRD GOLD data contains mother's characteristics, condition diagnoses, symptoms and prescriptions. Symptoms and diagnoses are recorded using ‘read codes', a hierarchical system which can be mapped to the International Classification of Diseases, tenth edition (ICD-10) and administrative codes. Information on antenatal care and birth details, pregnancy outcomes and postpartum care are recorded in addition to information on smoking status and substance use disorder.

To explore associations with deprivation and ethnicity, a subset of the CPRD data was linked with both Lower Super Output Area (LSOA) data and the Hospital Episodes Statistics (HES) database, which is the commissioned minimum data set for all secondary care providers in England. The English Index of Multiple Deprivation (IMD) consists of 37 indicators for deprivation across seven domains. Areas are ranked and divided into quintiles from least to most deprived.^17^ Ethnicity data were categorised into the Office for National Statistics definitions of ethnicity.

### Study population

Women who had given birth were defined using the CPRD Gold Pregnancy Register created by an algorithm developed jointly by CPRD and the London School of Hygiene and Tropical Medicine.^18^ All women with a pregnancy end date and live birth recorded between 1995 and 2020 and registered at a GP Practice that contributed to the CPRD database with a minimum of two years post pregnancy data were included. Women who experienced a stillbirth or termination of pregnancy were excluded. Any women recorded with a pregnancy end date on the 1st of January and a start of pregnancy data of the 27th of March (exactly 40 weeks earlier) were also excluded as this is expected to be an error where the true date of birth was unknown. Women with a pregnancy duration of less than 24 weeks or more than 43 weeks were excluded on the assumption these were substantially erroneous. Women who gave birth after 2018 were excluded from main analysis as second postpartum year follow-up data were not available for analysis.

Women were followed for two years after birth to determine prevalence and incidence of mental illness during this period. Women were followed up for less than two years if they gave birth after 2018 (less than two years of available data), moved GP Practice or died.

### Choice of primary outcome measure

The primary outcome measure was prevalence and incidence of mental illness likely to be eligible for specialist NHS PMH service care in the second postpartum year. It was important to ascertain cases that had continued into the second year or were of new onset after the first year. Incidence and prevalence of mental illness were explored over the study period. Subsequent years were also explored but not described here. Three fields (categories) were used to classify mental illness: diagnosis of a mental illness during consultations, symptom of mental illness recorded during consultations and prescription of psychotropic medications. Mental illnesses usually treated within specialist NHS PMH services were included: depression, anxiety (including post-traumatic stress disorder and obsessive compulsive disorder), bipolar disorder and affective psychosis (including puerperal psychosis), non-affective psychosis and personality disorder. Excluded conditions were sole diagnosis of eating disorders, neurodevelopmental conditions and substance use disorder as women are usually under alternative specialist services rather than PMH services.

Diagnostic and symptom codes to define mental illnesses were identified using previously published lists in relevant existing literature, clinicalcodes.org, or by searching the read code list using relevant strings (available at https://phenotypes.healthdatagateway.org/phenotypes). Symptom codes, such as ‘low mood’ and ‘anxiousness’ were mapped to relevant diagnostic categories. Medications were extracted using British National Formulary (BNF) chapters and each class of medication was assigned to their primary indication: antidepressants, antipsychotics, anxiolytics or hypnotics; mood stabilisers. Two perinatal consultant psychiatrists (GB, JJ) reviewed the read codes used to select cases. Cases were identified if women had any two of either a symptom, diagnosis or prescription code indicating a moderate to severe mental illness likely to need input from specialist PMH services to produce cautious estimates. To explore prevalence and incidence for each mental illness, only diagnosis and symptom codes were used due to potential overlap of medication treatment.

### Statistical methods

Characteristics of women, number of pregnancies, medical history and year of giving birth were described by presence of mental illness in the second postpartum year. Categorical variables were described using frequencies and percentages. Continuous variables were described by mean and standard deviation or median and interquartile range, as appropriate.

Prevalence and incidence of mental illness in the year preceding pregnancy, during and up to two years after pregnancy was described for the whole study period and by year of giving birth. Prevalence of mental illness refers to any mental illness reported in the second postpartum year after pregnancy. To estimate new cases of mental illness that started in the second year, incidence of mental illness that was not previously reported during pregnancy or the first year was described.

To assess for an increase in the prevalence of mental illness in the second postpartum year, a negative binomial regression model was used, a rate ratio and 95% confidence interval were reported.

Factors associated with mental illness in the second postpartum year were explored using unadjusted and adjusted logistic regression models accounting for multiple pregnancies by including random effects for women. The following variables were adjusted for in the logistic regression model: year of giving birth, mother's age, number of pregnancies, gestational age at birth, pre-pregnancy history of mental illness, diabetes (type 1, type 2 or gestational diabetes), smoking and substance use disorder. For the subset of data which were linked to LSOA and HES data, maternal ethnicity and IMD were also adjusted for in the logistic regression model. Odds ratios and 95% confidence intervals were reported.

All analysis was performed in Stata v18.0.

### Ethical approval

The study was approved by the Independent Scientific Advisory Committee for CPRD research (07/05/2021 21_000399). Informed consent was not required due to using de-identified patient data.

### Role of funding source

This study was funded by National Institute for Health and Care Research (NIHR) Applied Research Collaboration (ARC) West Midlands (NIHR200165) and had no role in the study design, data collection and analysis, interpretations of data, preparation of the manuscript, or decision to publish.

## Results

**Fig. 1:**
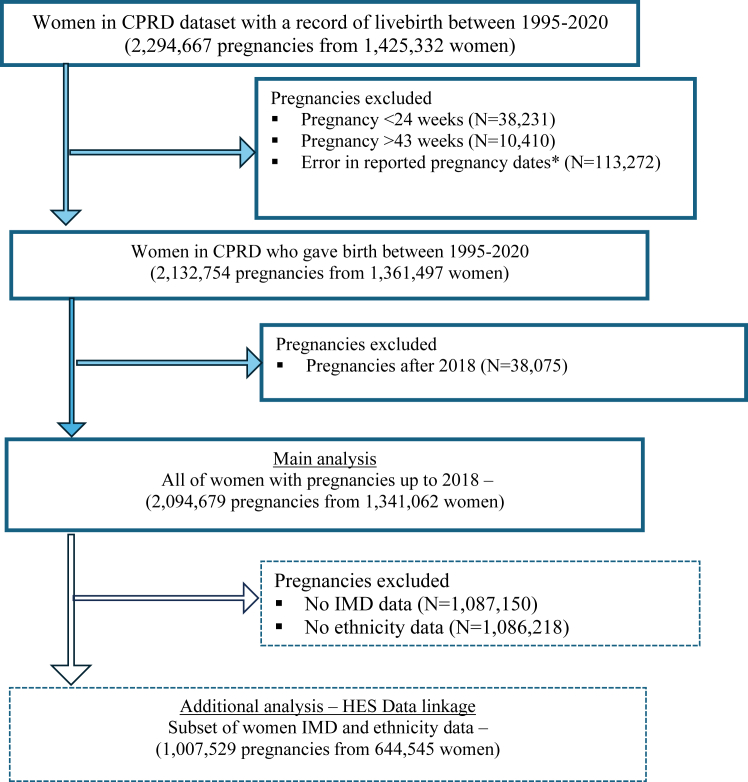
**Flow diagram**. ∗Error in pregnancy dates refers to pregnancies with an end date of the 1st of January and start date of 27th March.

The final study population included 2,132,754 pregnancies from 1,361,497 women who gave birth between 1995 and 2020 [Fig. 1]. Data were summarised for pregnancies up to 2018 allowing for two years of data after pregnancy. Up to 2018, there were 2,094,679 pregnancies from 1,341,062 women. Characteristics of the women and pregnancies were described according to the presence of mental illness during the second postpartum year [Table 1] and the subset of data linked to secondary care for ethnicity and IMD variables [Table 2].

**Table 1:** Baseline characteristics by the presence of mental illness during the second postpartum year.

Characteristics	No mental illness during the second postpartum year (n = 1,971,169)	Mental illness during the second postpartum year (n = 123,510)	Total (n = 2,094,679)
**Maternal age (years)**			
11–15	6278 (0.3%)	295 (0.2%)	6573 (0.3%)
16–19	146,553 (7.4%)	11,154 (9.0%)	157,707 (7.5%)
20–24	378,457 (19.2%)	29,309 (23.7%)	407,766 (19.5%)
25–29	544,776 (27.6%)	33,760 (27.3%)	578,536 (27.6%)
30–34	557,501 (28.3%)	30,051 (24.3%)	587,552 (28.0%)
35–39	277,479 (14.1%)	15,240 (12.3%)	292,719 (14.0%)
40+	60,125 (3.1%)	3701 (3.0%)	63,826 (3.0%)
**Number of pregnancies**			
1	306,557 (15.6%)	14,512 (11.7%)	321,069 (15.3%)
2	512,316 (26.0%)	27,131 (22.0%)	539,447 (25.8%)
3	432,567 (21.9%)	26,045 (21.1%)	458,612 (21.9%)
4	296,394 (15.0%)	19,801 (16.0%)	316,195 (15.1%)
5	183,347 (9.3%)	13,748 (11.1%)	197,095 (9.4%)
6	105,564 (5.4%)	8840 (7.2%)	114,404 (5.5%)
7	59,765 (3.0%)	5279 (4.3%)	65,044 (3.1%)
8	33,282 (1.7%)	3330 (2.7%)	36,612 (1.7%)
9	18,325 (0.9%)	1991 (1.6%)	20,316 (1.0%)
10	10,242 (0.5%)	1182 (1.0%)	11,424 (0.5%)
11	5995 (0.3%)	701 (0.6%)	6696 (0.3%)
12+	6815 (0.3%)	950 (0.8%)	7765 (0.4%)
**Duration of pregnancy (gestation, weeks)**			
Preterm (24–<34)	26,930 (1.4%)	2426 (2.0%)	29,356 (1.4%)
Near term (34–36)	63,458 (3.2%)	5845 (4.7%)	69,303 (3.3%)
Term (37–40)	1,685,939 (85.5%)	101,251 (82.0%)	1,787,190 (85.3%)
Post-term (41–43)	194,842 (9.9%)	13,988 (11.3%)	208,830 (10.0%)
**History of mental illness**^a^	60,984 (3.1%)	21,512 (17.4%)	82,496 (3.9%)
**Mental illness during pregnancy**	24,777 (1.3%)	11,048 (9.0%)	35,825 (1.7%)
**Mental illness during first postpartum year**	107,274 (5.4%)	50,020 (40.5%)	157,294 (7.5%)
**Diabetes (type 1, type 2, gestational)**^b^	157,027 (8.0%)	11,825 (9.6%)	168,852 (8.1%)
**Smoking**^b^	266,845 (13.5%)	32,076 (26.0%)	298,921 (14.3%)
**Substance use disorder**^b^	26,610 (1.3%)	4391 (3.6%)	31,001 (1.5%)
**Year of giving birth**			
1995	83,113 (4.2%)	2643 (2.1%)	85,756 (4.1%)
1996	85,787 (4.4%)	2955 (2.4%)	88,742 (4.2%)
1997	88,135 (4.5%)	3087 (2.5%)	91,222 (4.4%)
1998	90,422 (4.6%)	3491 (2.8%)	93,913 (4.5%)
1999	90,102 (4.6%)	3909 (3.2%)	94,011 (4.5%)
2000	90,298 (4.6%)	4651 (3.8%)	94,949 (4.5%)
2001	91,380 (4.6%)	5267 (4.3%)	96,647 (4.6%)
2002	92,076 (4.7%)	5878 (4.8%)	97,954 (4.7%)
2003	97,246 (4.9%)	6303 (5.1%)	103,549 (4.9%)
2004	99,547 (5.1%)	6550 (5.3%)	106,097 (5.1%)
2005	98,034 (5.0%)	6662 (5.4%)	104,696 (5.0%)
2006	100,095 (5.1%)	6759 (5.5%)	106,854 (5.1%)
2007	100,450 (5.1%)	6888 (5.6%)	107,338 (5.1%)
2008	99,439 (5.0%)	7365 (6.0%)	106,804 (5.1%)
2009	95,400 (4.8%)	7299 (5.9%)	102,699 (4.9%)
2010	93,368 (4.7%)	6988 (5.7%)	100,356 (4.8%)
2011	88,527 (4.5%)	6790 (5.5%)	95,317 (4.6%)
2012	83,918 (4.3%)	6317 (5.1%)	90,235 (4.3%)
2013	73,534 (3.7%)	5469 (4.4%)	79,003 (3.8%)
2014	64,723 (3.3%)	4782 (3.9%)	69,505 (3.3%)
2015	54,063 (2.7%)	4101 (3.3%)	58,164 (2.8%)
2016	43,845 (2.2%)	3540 (2.9%)	47,385 (2.3%)
2017	36,518 (1.9%)	3343 (2.7%)	39,861 (1.9%)
2018	31,149 (1.6%)	2473 (2.0%)	33,622 (1.6%)

a Mental illness within one year prior to pregnancy.

b First diagnosis prior to pregnancy.

**Table 2:** Baseline characteristics for subset of data by the presence of mental illness during the second postpartum year including ethnicity and IMD.

Characteristics	No mental illness during the second postpartum year (n = 946,681)	Mental illness during the second postpartum year (n = 60,848)	Total (n = 1,007,529)
**Mother's age (years)**			
11–15	2818 (0.3%)	159 (0.3%)	2977 (0.3%)
16–19	66,150 (7.0%)	5334 (8.8%)	71,484 (7.1%)
20–24	175,427 (18.5%)	14,240 (23.4%)	189,667 (18.8%)
25–29	258,424 (27.3%)	16,559 (27.2%)	274,983 (27.3%)
30–34	274,032 (28.9%)	14,967 (24.6%)	288,999 (28.7%)
35–39	139,652 (14.8%)	7730 (12.7%)	147,382 (14.6%)
40+	30,178 (3.2%)	1859 (3.1%)	32,037 (3.2%)
**Number of pregnancies**			
1	143,536 (15.2%)	6942 (11.4%)	150,478 (14.9%)
2	239,492 (25.3%)	12,675 (20.8%)	252,167 (25.0%)
3	206,249 (21.8%)	12,651 (20.8%)	218,900 (21.7%)
4	144,355 (15.2%)	9776 (16.1%)	154,131 (15.3%)
5	91,080 (9.6%)	6993 (11.5%)	98,073 (9.7%)
6	52,942 (5.6%)	4502 (7.4%)	57,444 (5.7%)
7	30,259 (3.2%)	2707 (4.4%)	32,966 (3.3%)
8	17,108 (1.8%)	1865 (3.1%)	18,973 (1.9%)
9	9750 (1.0%)	1107 (1.8%)	10,857 (1.1%)
10	5281 (0.6%)	665 (1.1%)	5946 (0.6%)
11	3109 (0.3%)	398 (0.7%)	3507 (0.3%)
12+	3520 (0.4%)	567 (0.9%)	4087 (0.4%)
**Length of pregnancy (gestation, weeks)**			
Premature	15,145 (1.6%)	1346 (2.2%)	16,491 (1.6%)
Near term	35,695 (3.8%)	3144 (5.2%)	38,839 (3.9%)
Term	780,512 (82.4%)	48,468 (79.7%)	828,980 (82.3%)
Post-term	115,329 (12.2%)	7890 (13.0%)	123,219 (12.2%)
**History of mental illness**^a^	31,001 (3.3%)	10,667 (17.5%)	41,668 (4.1%)
**Mental illness during pregnancy**	12,643 (1.3%)	5534 (9.1%)	18,177 (1.8%)
**Mental illness during first postpartum year**	52,069 (5.5%)	25,003 (41.1%)	77,072 (7.7%)
**Diabetes (type 1, type 2 or gestational)**^b^	79,196 (8.4%)	5565 (9.1%)	84,761 (8.4%)
**Smoking**^b^	129,277 (13.7%)	14,781 (24.3%)	144,058 (14.3%)
**Substance use disorder**^b^	9659 (1.0%)	1843 (3.0%)	11,502 (1.1%)
**IMD, quintiles**			
1 (least deprived)	205,464 (21.7%)	9961 (16.4%)	215,425 (21.4%)
2	187,354 (19.8%)	10,612 (17.4%)	197,966 (19.6%)
3	183,095 (19.3%)	11,743 (19.3%)	194,838 (19.3%)
4	184,082 (19.4%)	13,323 (21.9%)	197,405 (19.6%)
5 (most deprived)	186,686 (19.7%)	15,209 (25.0%)	201,895 (20.0%)
**Ethnicity**			
Bangladeshi	5928 (0.6%)	144 (0.2%)	6072 (0.6%)
Black African	23,320 (2.5%)	339 (0.6%)	23,659 (2.3%)
Black Caribbean	8139 (0.9%)	283 (0.5%)	8422 (0.8%)
Black Other	6635 (0.7%)	187 (0.3%)	6822 (0.7%)
Chinese	5923 (0.6%)	67 (0.1%)	5990 (0.6%)
Indian	20,678 (2.2%)	393 (0.6%)	21,071 (2.1%)
Mixed	11,198 (1.2%)	594 (1.0%)	11,792 (1.2%)
Other Asian	14,451 (1.5%)	315 (0.5%)	14,766 (1.5%)
Other	21,874 (2.3%)	632 (1.0%)	22,506 (2.2%)
Pakistani	18,791 (2.0%)	564 (0.9%)	19,355 (1.9%)
Unknown	30,090 (3.2%)	842 (1.4%)	30,932 (3.1%)
White	779,654 (82.4%)	56,488 (92.8%)	836,142 (83.0%)
**Year of giving birth**			
1995	32,955 (3.5%)	1313 (2.2%)	34,268 (3.4%)
1996	36,209 (3.8%)	1516 (2.5%)	37,725 (3.7%)
1997	40,079 (4.2%)	1645 (2.7%)	41,724 (4.1%)
1998	43,103 (4.6%)	1912 (3.1%)	45,015 (4.5%)
1999	43,388 (4.6%)	2165 (3.6%)	45,553 (4.5%)
2000	44,607 (4.7%)	2588 (4.3%)	47,195 (4.7%)
2001	45,292 (4.8%)	2835 (4.7%)	48,127 (4.8%)
2002	46,212 (4.9%)	3229 (5.3%)	49,441 (4.9%)
2003	49,216 (5.2%)	3387 (5.6%)	52,603 (5.2%)
2004	50,809 (5.4%)	3453 (5.7%)	54,262 (5.4%)
2005	50,456 (5.3%)	3510 (5.8%)	53,966 (5.4%)
2006	51,671 (5.5%)	3569 (5.9%)	55,240 (5.5%)
2007	52,335 (5.5%)	3687 (6.1%)	56,022 (5.6%)
2008	51,716 (5.5%)	3962 (6.5%)	55,678 (5.5%)
2009	49,428 (5.2%)	3783 (6.2%)	53,211 (5.3%)
2010	48,128 (5.1%)	3584 (5.9%)	51,712 (5.1%)
2011	44,841 (4.7%)	3436 (5.6%)	48,277 (4.8%)
2012	42,287 (4.5%)	3074 (5.1%)	45,361 (4.5%)
2013	35,876 (3.8%)	2368 (3.9%)	38,244 (3.8%)
2014	29,095 (3.1%)	1918 (3.2%)	31,013 (3.1%)
2015	22,030 (2.3%)	1396 (2.3%)	23,426 (2.3%)
2016	15,849 (1.7%)	1006 (1.7%)	16,855 (1.7%)
2017	11,917 (1.3%)	914 (1.5%)	12,831 (1.3%)
2018	9182 (1.0%)	598 (1.0%)	9780 (1.0%)

a History of mental illness 1 year prior to pregnancy.

b First diagnosis prior to pregnancy.

### Prevalence and incidence of mental illness in the second postpartum year

122,330 diagnosis codes, 80,470 symptom codes and 262,806 prescription codes were used to identify mental illness in the second postpartum year (Supplementary Material 1). After selecting cases by combining any two of diagnosis, symptom or prescription code, the prevalence of mental illness in the second postpartum year increased over the study period from 3.1% in 1995 to 7.4% in 2018, peaking at 8.4% in 2017 [Table 3]. The negative binomial model showed a significant increase in rate ratios for each year following 1995, suggesting an increase in prevalence of mental illness over the study period. After excluding previous mental illness during pregnancy and the first postpartum year, the incidence of mental health illness in the second postpartum year (representing new onset cases) increased from 1.9% in 1995 to 3.8% in 2018 [Table 3]. Across the study period, the prevalence of mental illness in the second postpartum year was 5.9% and the incidence was 3.3%, therefore, new onset cases represented 56.6% of all cases in the second postpartum year. The comparable prevalence for the first year from this dataset was 7.5% with 6.6% being new onset [Table 3].

**Table 3:** Prevalence and incidence of mental illness (combination of any two of diagnosis, symptom, or prescription code) by year of birth and timing in relation to pregnancy.

Year	Number of pregnancies	Pre-pregnancy no (%)^a^	During pregnancy no (%)	Prevalence post-pregnancy		Incidence^b^ post-pregnancy	
				0–1 year no (%)	1–2 years no (%)	0–1 years no (%)	1–2 year no (%)
1995	85,756	1315 (1.5)	487 (0.6)	3300 (3.8)	2643 (3.1)	3100 (3.6)	1630 (1.9)
1996	88,742	1629 (1.8)	559 (0.6)	3694 (4.2)	2955 (3.3)	3453 (3.9)	1770 (2.0)
1997	91,222	1753 (1.9)	628 (0.7)	4080 (4.5)	3087 (3.4)	3801 (4.2)	1791 (2.0)
1998	93,913	1975 (2.1)	721 (0.8)	4265 (4.5)	3491 (3.7)	3945 (4.2)	2152 (2.3)
1999	94,011	2143 (2.3)	788 (0.8)	4637 (4.9)	3909 (4.2)	4290 (4.6)	2375 (2.5)
2000	94,949	2148 (2.3)	905 (1.0)	5718 (6.0)	4651 (4.9)	5302 (5.6)	2797 (2.9)
2001	96,647	2483 (2.6)	1086 (1.1)	6537 (6.8)	5267 (5.4)	6011 (6.2)	3010 (3.1)
2002	97,954	3001 (3.1)	1333 (1.4)	7445 (7.6)	5878 (6.0)	6818 (7.0)	3271 (3.3)
2003	103,549	3647 (3.5)	1691 (1.6)	8228 (7.9)	6303 (6.1)	7439 (7.2)	3457 (3.3)
2004	106,097	4136 (3.9)	1833 (1.7)	8249 (7.8)	6550 (6.2)	7399 (7.0)	3663 (3.5)
2005	104,696	4250 (4.1)	1787 (1.7)	8485 (8.1)	6662 (6.4)	7634 (7.3)	3799 (3.6)
2006	106,854	4252 (4.0)	1795 (1.7)	9087 (8.5)	6759 (6.3)	8265 (7.7)	3842 (3.6)
2007	107,338	4468 (4.2)	1871 (1.7)	8877 (8.3)	6888 (6.4)	8038 (7.5)	3943 (3.7)
2008	106,804	4846 (4.5)	1978 (1.9)	9162 (8.6)	7365 (6.9)	8281 (7.8)	4155 (3.9)
2009	102,699	4868 (4.7)	2059 (2.0)	9118 (8.9)	7299 (7.1)	8188 (8.0)	4145 (4.0)
2010	100,356	4889 (4.9)	2075 (2.1)	8906 (8.9)	6988 (7.0)	7952 (7.9)	3984 (4.0)
2011	95,317	5094 (5.3)	2304 (2.4)	8570 (9.0)	6790 (7.1)	7560 (7.9)	3834 (4.0)
2012	90,235	5158 (5.7)	2302 (2.6)	8075 (8.9)	6317 (7.0)	7095 (7.9)	3541 (3.9)
2013	79,003	4499 (5.7)	1946 (2.5)	7003 (8.9)	5469 (6.9)	6146 (7.8)	3042 (3.9)
2014	69,505	4008 (5.8)	1880 (2.7)	6348 (9.1)	4782 (6.9)	5488 (7.9)	2582 (3.7)
2015	58,164	3580 (6.2)	1631 (2.8)	5438 (9.3)	4101 (7.1)	4711 (8.1)	2220 (3.8)
2016	47,385	3030 (6.4)	1549 (3.3)	4581 (9.7)	3540 (7.5)	3884 (8.2)	1846 (3.9)
2017	39,861	2917 (7.3)	1389 (3.5)	4039 (10.1)	3343 (8.4)	3385 (8.5)	1792 (4.5)
2018	34,098	2452 (7.2)	1245 (3.7)	3497 (10.3)	2473 (7.4)	2969 (8.7)	1285 (3.8)
2019	28,417	2237 (7.9)	1106 (3.9)	2690 (9.6)	–	2293 (8.1)	–
2020	9182	788 (8.6)	390 (4.2)	–	–	–	–
Total	2,132,754	37,338 (4.0)	37,338 (1.8)	160,029 (7.5)	123,510 (5.9)	141,154 (6.6)	69,926 (3.3)

a Pre-pregnancy refers to any mental health illness in the year before pregnancy.

b Incidence of mental illness (combination of any two of the following: diagnosis, symptom or prescription code) excluding any women with mental illness during pregnancy or the preceding postpartum years.

### Type of mental illness in the second postpartum year

**Fig. 2:**
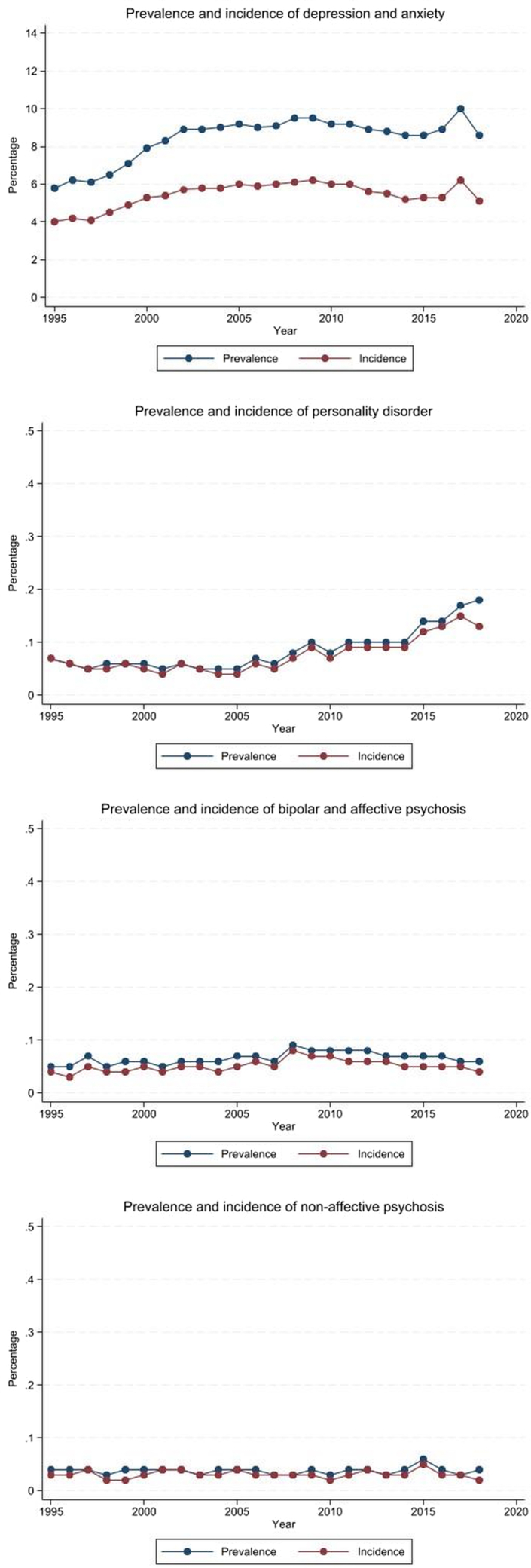
Prevalence and incidence of mental health illness by type of diagnosis and symptoms.

The most common prevalent mental illness type in the second year (using diagnosis and symptom code only) was depression and anxiety (8.4%). Personality disorder (0.08%), bipolar and affective psychosis (0.07%) and non-affective psychosis 0.04%) were all uncommon [Table 4]. Whilst cases of depression and anxiety peaked in 2017, cases of personality disorder continued to rise from 0.07% in 1995 to 0.18% in 2018 and cases of non-affective psychosis and bipolar and affective psychosis have remained stable throughout the study period [Fig. 2].

**Table 4:** Prevalence and incidence of each mental illness by timing in relation to pregnancy over the study period 1995–2018.

Mental illness type	Pre-pregnancy no (%)	During pregnancy no (%)	Prevalence post-pregnancy no (%)		Incidence post-pregnancy no (%)	
			0–1 years	1–2 years	0–1 years	1–2 year
Depression and anxiety	141,299 (6.6)	79,272 (3.7)	241,484 (11.4)	176,003 (8.4)	210,983 (10.3)	98,347 (5.4)
Personality disorder	1680 (0.08)	976 (0.05)	1523 (0.07)	1598 (0.08)	1021 (0.05)	642 (0.03)
Non-affective psychosis	723 (0.03)	514 (0.02)	1119 (0.05)	790 (0.04)	1021 (0.05)	642 (0.03)
Bipolar and affective psychosis	1144 (0.05)	918 (0.04)	1830 (0.09)	1377 (0.07)	1665 (0.08)	1099 (0.05)

The most common incident mental illness type in the second postpartum year was depression and anxiety (5.4%), followed by bipolar disorder and affective psychosis (0.05%), non-affective psychosis (0.03%) and personality disorder (0.03%) [Table 4]. Incident cases of depression and anxiety peaked in 2009 and 2017, cases of personality disorder consistently increased throughout the study period from 0.07 in 1995 to 0.13 in 2018 [Fig. 2]. Non-affective psychosis and bipolar and affective psychoses cases remained stable throughout the study period.

### Factors associated with mental illness

For the unadjusted analysis, all factors investigated were associated with mental illness during the second postpartum year [Table 5]. For the adjusted analysis, the odds of having a mental illness were higher for women in lower age groups compared to 30–34 years old (reference chosen as average age for birth) and the odds were lower for women aged 35–39 (OR 0.97, 95% CI: 0.95, 1.00).

**Table 5:** Logistic regression models exploring associations between mental illness during the second postpartum year and characteristics.

	Unadjusted odds ratio (95% CI)	Adjusted odds ratio (95% CI)
**Maternal age, years**		
30–34	Reference	Reference
11–15	0.76 (0.67–0.88)	1.18 (1.04–1.33)
16–19	1.33 (1.30–1.37)	1.38 (1.35–1.42)
20–24	1.41 (1.38–1.44)	1.26 (1.24–1.28)
25–29	1.14 (1.12–1.17)	1.10 (1.08–1.12)
35–39	1.03 (1.08–1.06)	0.97 (0.95–1.00)
40+	1.17 (1.29–1.22)	1.03 (0.99–1.07)
**Number of pregnancies**		
1	Reference	Reference
2	1.15 (1.12–1.17)	1.16 (1.13–1.19)
3	1.34 (1.30–1.37)	1.29 (1.26–1.32)
4	1.52 (1.47–1.56)	1.38 (1.35–1.41)
5	1.76 (1.71–1.82)	1.48 (1.44–1.52)
6	2.02 (1.95–2.09)	1.57 (1.52–1.62)
7	2.16 (2.06–2.26)	1.64 (1.58–1.71)
8	2.54 (2.40–2.69)	1.78 (1.70–1.86)
9	2.81 (2.61–3.03)	1.85 (1.75–1.96)
10	3.10 (2.81–3.42)	1.94 (1.80–2.09)
11	3.21 (2.83–3.64)	1.90 (1.72–2.09)
12+	3.97 (3.53–4.47)	2.17 (1.99–2.36)
**Duration of pregnancy (gestation, weeks)**		
Term (37–40 weeks)	Reference	Reference
Premature (24–33 weeks)	1.56 (1.48–1.64)	1.21 (1.15–1.27)
Near term (34–36 weeks)	1.60 (1.54–1.65)	1.21 (1.17–1.25)
Post-term (41–43 weeks)	1.22 (1.20–1.25)	1.07 (1.04–1.09)
**History of mental illness**^a^	6.33 (6.20–6.46)	2.46 (2.41–2.52)
**Mental illness during pregnancy**	7.52 (7.29–7.76)	1.99 (1.93–2.05)
**Mental illness during first postpartum year**	12.92 (12.73–13.12)	9.01 (8.88–9.15)
**Diabetes (type 1, type 2, gestational)**	1.29 (1.26–1.32)	1.00 (0.98–1.03)
**Smoking**	2.53 (2.48–2.57)	1.37 (1.35–1.39)
**Substance use disorder**	3.43 (3.28–3.59)	1.54 (1.48–1.60)
**Year of giving birth**		
1995	Reference	Reference
1996	1.09 (1.03–1.16)	1.05 (0.99–1.11)
1997	1.11 (1.05–1.18)	1.04 (0.98–1.10)
1998	1.26 (1.19–1.34)	1.14 (1.08–1.21)
1999	1.44 (1.36–1.52)	1.26 (1.20–1.33)
2000	1.77 (1.68–1.88)	1.44 (1.37–1.52)
2001	2.03 (1.92–2.14)	1.57 (1.49–1.65)
2002	2.29 (2.17–2.42)	1.66 (1.58–1.75)
2003	2.35 (2.22–2.48)	1.64 (1.56–1.72)
2004	2.39 (2.27–2.53)	1.65 (1.57–1.73)
2005	2.52 (2.39–2.66)	1.66 (1.58–1.74)
2006	2.51 (2.38–2.65)	1.59 (1.52–1.67)
2007	2.58 (2.44–2.72)	1.62 (1.55–1.71)
2008	2.82 (2.67–2.97)	1.72 (1.64–1.80)
2009	2.95 (2.79–3.11)	1.75 (1.67–1.84)
2010	2.87 (2.72–3.03)	1.70 (1.62–1.78)
2011	2.99 (2.83–3.15)	1.73 (1.64–1.82)
2012	2.9 (2.75–3.06)	1.68 (1.60–1.77)
2013	2.88 (2.72–3.05)	1.67 (1.59–1.76)
2014	2.85 (2.69–3.02)	1.63 (1.55–1.72)
2015	2.95 (2.78–3.13)	1.67 (1.58–1.76)
2016	3.16 (2.97–3.36)	1.77 (1.67–1.87)
2017	3.67 (3.45–3.91)	2.00 (1.89–2.12)
2018	3.07 (2.87–3.28)	1.70 (1.60–1.81)

a History of mental health illness 1 year prior to pregnancy.

The odds of having a mental illness increased with each additional pregnancy: the odds were 16% higher (95% CI: 1.13, 1.19) for mothers with two pregnancies compared to mothers with one pregnancy and odds continued to increase.

The odds of having mental illness were 21% higher for mothers who gave birth preterm (95% CI: 1.15, 1.27), 21% higher for near-term (95% CI: 1.17, 1.25) and 7% higher for post-term (95% CI: 1.04, 1.09) compared to term births.

The odds of mothers having mental illness were higher if women had the following conditions prior to pregnancy: previous mental illness in the year before pregnancy (OR 2.46, 95% CI: 2.41, 2.52), mental illness during pregnancy (OR 1.99, 95% CI: 1.93, 2.05), mental illness during the first postpartum year (OR 9.01, 95% CI: 8.88, 9.15), being a smoker (OR 1.37, 95% CI: 1.35, 1.39 and women with substance use disorders (OR 1.54, 95% CI: 1.48, 1.60).

The odds of having mental illness were higher for women who gave birth in every year after 1995 compared to 1995 births.

Factors associated with women having mental illness including IMD and ethnicity variables from the subset of data linked to LSOA and HES showed similar results for the factors in the main analysis [Table 6]. The odds of being recorded as having mental illness were higher for women in the most deprived quintile compared to the least deprived quintile (OR 1.29, 95% CI: 1.25, 1.33) and were lower for women of every ethnicity compared to mothers of White ethnicity. For example, the odds of having a mental illness were 61% lower for women of Indian ethnicity compared to White ethnicity (95% CI: 0.35–0.43).

**Table 6:** Logistic regression models exploring associations between mental health illness during the second postpartum year and characteristics including subset with IMD and ethnicity variables.

	Unadjusted odds ratio (95% CI) p-value	Adjusted odds ratio (95% CI) p-value
**Mother's age, years**		
30–34	Reference	Reference
11–15	0.93 (0.77–1.13)	1.17 (0.98–1.38)
16–19	1.41 (1.36–1.47)	1.23 (1.18–1.27)
20–24	1.47 (1.43–1.51)	1.18 (1.15–1.21)
25–29	1.17 (1.14–1.20)	1.09 (1.06–1.12)
35–39	1.02 (0.99–1.06)	0.98 (0.95–1.01)
40+	1.15 (1.08–1.22)	1.02 (0.97–1.08)
**Number of pregnancies**		
1	Reference	Reference
2	1.12 (1.08–1.16)	1.11 (1.08–1.15)
3	1.34 (1.29–1.39)	1.26 (1.22–1.30)
4	1.50 (1.44–1.56)	1.33 (1.29–1.38)
5	1.77 (1.69–1.85)	1.44 (1.39–1.50)
6	2.00 (1.90–2.11)	1.50 (1.44–1.57)
7	2.13 (2.00–2.27)	1.56 (1.48–1.65)
8	2.74 (2.54–2.96)	1.77 (1.67–1.89)
9	2.90 (2.63–3.21)	1.77 (1.63–1.91)
10	3.33 (2.92–3.80)	1.89 (1.71–2.09)
11	3.52 (2.97–4.16)	1.87 (1.65–2.13)
12+	4.50 (3.86–5.26)	2.25 (2.00–2.52)
**Duration of pregnancy**		
Term (37–40 weeks)	Reference	Reference
Premature (24–33 weeks)	1.48 (1.38–1.59)	1.16 (1.08–1.23)
Near term (34–36 weeks)	1.46 (1.40–1.53)	1.15 (1.10–1.20)
Post-term (41–43 weeks)	1.12 (1.09–1.15)	1.01 (0.99–1.04)
**History of mental health illness**^a^	5.93 (5.76–6.11)	2.32 (2.25–2.39)
**Mental illness during pregnancy**	7.12 (6.82–7.43)	1.91 (1.83–1.99)
**Mental illness during first postpartum year**	13.05 (12.77–13.33)	8.83 (8.64–9.03)
**Diabetes (type 1, type 2, gestational)**	1.14 (1.10–1.19)	1.02 (0.99–1.06)
**Smoking**	2.23 (2.17–2.28)	1.24 (1.21–1.27)
**Substance use disorder**	3.88 (3.61–4.18)	1.53 (1.44–1.62)
**IMD quintile**		
1 (least deprived)	Reference	Reference
2	1.19 (1.15–1.24)	1.09 (1.05–1.12)
3	1.38 (1.33–1.43)	1.15 (1.11–1.18)
4	1.59 (1.54–1.65)	1.24 (1.20–1.27)
5 (most deprived)	1.84 (1.78–1.90)	1.29 (1.25–1.33)
**Ethnicity**		
White	Reference	Reference
Bangladeshi	0.28 (0.23–0.34)	0.40 (0.34–0.48)
Black African	0.17 (0.15–0.19)	0.28 (0.25–0.31)
Black Caribbean	0.43 (0.38–0.50)	0.57 (0.50–0.64)
Black Other	0.33 (0.28–0.39)	0.48 (0.41–0.55)
Chinese	0.12 (0.10–0.16)	0.24 (0.19–0.31)
Indian	0.22 (0.19–0.24)	0.39 (0.35–0.43)
Mixed	0.70 (0.63–0.78)	0.76 (0.69–0.83)
Other Asian	0.25 (0.22–0.28)	0.41 (0.36–0.46)
Other	0.35 (0.31–0.38)	0.53 (0.49–0.58)
Pakistani	0.36 (0.32–0.40)	0.47 (0.43–0.52)
Unknown	0.33 (0.31–0.36)	0.58 (0.53–0.62)
**Year of giving birth**		
1995	Reference	Reference
1996	1.06 (0.97–1.16)	1.01 (0.93–1.09)
1997	1.04 (0.95–1.13)	0.97 (0.90–1.05)
1998	1.15 (1.06–1.25)	1.08 (1.00–1.16)
1999	1.32 (1.22–1.43)	1.20 (1.11–1.29)
2000	1.58 (1.46–1.71)	1.36 (1.26–1.46)
2001	1.75 (1.61–1.89)	1.41 (1.31–1.51)
2002	1.99 (1.84–2.15)	1.54 (1.43–1.65)
2003	1.97 (1.82–2.12)	1.47 (1.37–1.58)
2004	1.94 (1.8–2.1)	1.46 (1.36–1.57)
2005	2.02 (1.88–2.19)	1.48 (1.38–1.59)
2006	2.01 (1.86–2.17)	1.43 (1.33–1.53)
2007	2.07 (1.92–2.24)	1.47 (1.37–1.57)
2008	2.28 (2.12–2.46)	1.57 (1.47–1.68)
2009	2.29 (2.13–2.48)	1.54 (1.44–1.65)
2010	2.22 (2.06–2.4)	1.51 (1.41–1.62)
2011	2.32 (2.15–2.51)	1.53 (1.42–1.64)
2012	2.15 (1.99–2.33)	1.43 (1.33–1.54)
2013	1.92 (1.77–2.09)	1.31 (1.21–1.41)
2014	1.92 (1.76–2.09)	1.29 (1.19–1.39)
2015	1.82 (1.66–1.99)	1.21 (1.11–1.32)
2016	1.8 (1.63–1.99)	1.22 (1.11–1.34)
2017	2.24 (2.02–2.49)	1.48 (1.35–1.63)
2018	1.81 (1.61–2.04)	1.22 (1.09–1.36)

a History of mental health illness 1 year prior to pregnancy.

## Discussion

This study shows that prevalence of moderate to severe mental illness identified by GPs in a UK cohort of over 1.3 million women during the second postpartum year was 5.9% and increased over time from 1995. New onset cases represented 56.6% of all cases in the second postpartum year, hence recorded new onset cases were more common than those that continued from the first year. Given the lifelong gains of specialist perinatal mental health (PMH) care and interventions for both the mother and baby, findings highlight ongoing need, indicating that the extension PMH services to two years postpartum in England was justified.

Few studies have assessed prevalence of moderate to severe mental illness in the second postpartum year. A UK study^6^ using CPRD found a prevalence of mental illness in the mothers of children aged 0–2 of 21.9% between 2005 and 2007, higher than our study. However, this study included women with a mental illness in both the first and second postpartum year. In addition, they defined cases using only one measure of diagnosis code or referral code, or a combination of symptom and prescription code, whereas we defined cases only if women had any two of either a symptom, diagnosis or prescription code to indicate a mental illness more likely to require specialist PMH services; hence our estimates are lower despite similar individual frequencies prescription, symptom and diagnosis codes to Abel et al.^6^

Our study found that there was an increase in all mental illness since 1995. Apart from depression, which is most likely to have been more consistently reported by GPs within the study time period, increases in all other conditions may represent improvements in recognition and diagnosis over time, rather than a real time increase in these conditions.

Our study showed an association between preterm birth and PMI in the second postpartum year. This corresponds with a United States study which found that any maternal mental illness was associated with odds of birth less than 37 weeks' gestation (OR 1.45, 95% CI: 1.38, 1.52), less than 34 weeks' gestation (OR 1.47, 95% CI: 1.35, 1.59) and less than 28 weeks’ gestation (OR 1.57, 1.36,1.82).^19^ It is likely however that preterm birth also increases risk of maternal mental illness, in particular depression and anxiety.

That women with pre-existing mental illness were more likely to experience mental illness in the second year after giving birth is unsurprising and consistent with the existing literature.^5^ It is possible that women with a pre-existing PMI are more likely to be identified by a GP due to their mental health history. Women with mental illness prior to pregnancy are likely to require ongoing support from specialist PMH services and close monitoring of medication.

Women with a recorded history of smoking were more likely to have mental illness in second year after birth and this is also consistent with studies indicating that women with PMI are more likely to continue smoking during pregnancy than those without^20^ echoing general population trends of smoking. Given evidence that stopping smoking can improve mental health,^21^ and the effect of maternal smoking on infant health,^22^identification of effective smoking cessation strategies for women with PMI is crucial.

Our study found that women in the most deprived quintile were most likely to have PMI in the second year postpartum and this is consistent with existing literature suggesting that socioeconomic deprivation and risk of mental illness are positively related.^5^

Women from ethnic minority groups, particularly South Asian women were less likely to have PMI recorded in clinical records and therefore the prevalence in this group is likely to be an underestimate. This may stem from lack of awareness of perinatal mental disorders and cultural expectations of societal role of motherhood^23^ but also high levels of stigma, shame and fear of judgement commonly found among South Asian communities which prevents women from accessing mental health care.^24^^,^^25^ A UK population-based cohort study in 2017 found that women in Black African, Asian and White Other groups accessed community mental health services less than White British women but had higher incidence of involuntary admission than White British women.^26^ This suggests that there are inequities in access to early support and care for women in minority ethnic groups and that lower primary care access is not synonymous with low level of need. To address this, adaptations to interventions and culturally adapted therapies, led by a cultural experts, that acknowledge topics of race, ethnicity and culture within PMH services, with training and support for staff is essential.^27^, ^28^, ^29^

Interestingly, despite increasing evidence of an association between diabetes and perinatal mental illness,^30^^,^^31^ our study did not find a significant difference in mental illness in the second postpartum year and a recorded medical history of either type 1, type 2 or gestational diabetes. Risk of mental illness in the second postpartum year was higher for adolescent mothers compared to any other age group. There is a paucity of literature examining prevalence, experiences and effective treatment for adolescent mothers experiencing mental illness.^32^ Given that it is often associated with social determinants of health such as poverty and illiteracy,^33^ there is a need to understand the needs of adolescent mothers with mental illness, particularly in low and middle-income countries where there are higher rates of PMI and adolescent pregnancies.^34^

There are no known studies exploring prevalence of specific mental illnesses in the second postpartum. When comparing our first-year prevalence estimates of specific mental illnesses to existing studies, our prevalence estimates are slightly lower than others,^35^ although consistent with other studies exploring illnesses such as postnatal depression using primary care data.^36^ This is likely to be reflect lower reporting of mental illnesses in primary care.

This study used a large nationally representative primary care database over a considerable time period to describe prevalence and incidence of mental illness in pregnancy in the second postpartum year. It is the first known study to explore prevalence of GP identified mental illness likely that requires specialist PMH care in the second year postpartum using data that has high specificity and positive predictive value for detecting some mental health problems.^37^ Neither prevalence nor incidence in the second postpartum year were previously known despite the change in policy for provision of specialist services in England. Another strength is that two senior perinatal psychiatrists independently reviewed case selection codes and existing methodology was used to identify women who had given birth.

In terms of limitations, this study only included women who consulted a GP about their mental health and some women, even with moderate and severe mental illness, may not consult a GP. Some pregnancies are not identified using the CPRD pregnancy dataset register algorithm^18^ and therefore some pregnancies, which may have been complex pregnancies with an ‘unknown outcome’, will have been missed. If women left a CPRD practice before the two-year postpartum cutoff period, any subsequent new mental illness would have been missed. Due to dataset constraints, it was not possible to assess severity of mental illness definitively and determine exactly how many additional women with *moderate* rather than previously only *severe* illness may have benefitted from the extension of specialist NHS PMH services to include the second postpartum year. However, we did ensure that of the three possible categories, two fields were used (symptom, diagnosis or prescription) to define cases leading to a more conservative estimate and the likelihood that the cases selected were less likely to be mild cases of mental illness treated in primary care. We did not use ‘referrals’ to identify cases as it would not provide a reliable date for first diagnosis and therefore will not have identified cases where women had either a diagnosis or symptom code only and were referred for psychological therapy via the GP. In addition, there may have been some women inaccurately classed as incident cases in the second year due to initiation of pharmacotherapy, when they had already been referred for psychological therapy in the first year. There is no recognised definition distinguishing severe from moderate mental illness however, women with a diagnosis of personality disorder, non-affective psychosis and bipolar and affective psychosis in primary care data will generally have been diagnosed by a psychiatrist and therefore under the care of specialist PMH services. Associations with diverse factors were explored, though dataset limitations meant it was not possible to explore low social support, marital status and domestic abuse as variables in regression analysis which are known to be associated with PMI.^38^^,^^39^ It is possible that some second-year mental illness continued from the first but did not continue to be recorded in the GP records so the proportion of ongoing cases into the second year may be an underestimate. There may have been some loss to follow up due to women who moved GP practices in the two years after giving birth.

It was not our intention to explore specific subgroups of mental illness, such as post-traumatic stress disorder, and this may have identified important differences to explore in future research.

This study shows that among women who consulted their GP, PMI often does not resolve by 12 months postpartum and a large proportion of cases within the second postpartum year are new onset. The findings confirm the need for the policy extending NHS specialist PMH services to two years postpartum to improve outcomes for mothers and babies. Recent NHS data suggests that 57,000 new and expectant mothers have received specialist support for PMI in the last 12 months, an increase by over a third since 2022, supporting study findings that there is need and demand for specialist PMH services ranging from preconception to two years postpartum.^40^ These findings are applicable to other high-income countries with a similar perinatal mental health care services. Whilst it is recognised that some women with mild perinatal mental illness will not require specialist input, to ensure women with moderate and severe mental illness receive specialist care, it is crucial that clinicians, particularly GPs, and women are aware of continuing risk of mental illness in the second postpartum year, and the availability of specialist provision to improve long term maternal and infant health.

The long-term costs from lack of timely access to good quality perinatal mental health care in the UK are estimated to be £1.2 billion to the NHS and social services and £8.1 billon to society. The investment for specialist perinatal mental health services to two years totalled £365 million over a five-year period. Given the very large estimated costs of PMI,^2^ future research should investigate how to further define severity of perinatal mental illness so that resource can be appropriately attributed, particularly in countries with limited healthcare resources. For example, evidence shows that task sharing with non-specialist providers could be a solution for treatment of mental illnesses such as depression or anxiety,^10^^,^^41^ particularly in low and middle income countries facing shortage of mental health professionals. Alternatively, improving access to generic psychological therapies not specific to the perinatal period such as the UK's NHS Talking Therapies may offer a solution to treatment access, although their effectiveness in perinatal women is largely unknown.^11^ However, it is important to acknowledge that task sharing of psychosocial interventions, whilst important for improving access for those whose needs are unmet, should not be a substitute for existing mental health-care systems and that women with severe mental illness are still likely require support from specialist PMH services as the evidence on task sharing for severe mental illness is not well understood.^42^ Further research could explore prevalence of particular subgroups of mental illness, such as post-traumatic stress disorder and illness trajectories for women who have new onset in the second year. Further research should explore how to improve equity in access to PMH services in the second postpartum year by facilitating and building upon culturally adapted models for screening and treatment of PMI for ethnic minority women.

## Contributors

EJ, CM, BT, GB and JJ conceived the study. EJ and JRT wrote the protocol, EJ, JRT, GB and JJ developed read code lists to identify cases. JRT and LQ had direct access to the data, performed data linkage, data extraction and analysis and verified the underlying data reported. All authors supported with interpretation of findings. EJ wrote the manuscript and all authors edited and read the final draft.

## Data sharing statement

The study protocol and coding framework developed to identify cases are available on https://phenotypes.healthdatagateway.org/phenotypes/PH1683/version/3563/detail/.

## Declaration of interests

EJ, CM, BT and JJ report various grants from National Institute for Health Research (NIHR). CM, BT and LQ are funded in part by the West Midlands Applied Research Collaboration NIHR-WM ARC. CM is part of the NIHR Global Health Research Advisory Group. BT is Steering Group Member of NIHR BRACE programme and West Midlands Women's Health and Institute for Health Equity and Social Care and Institute Accelerator Board Member for University Hospitals Coventry and Warwickshire. JJ is Clinical lead for Midlands Perinatal Mental Health Clinical Network, member of the Pharmacological Therapies Committee within Birmingham and Solihull Mental Health Trust and has received honoraria for a lecture on psychotropic medication in the perinatal period at University of Exeter. JJ had a short contract with Niche Health and Social care Consulting Limited as subject matter expert, attended a training event by Medice UK and was previously academic secretary for Royal College of Psychiatrists perinatal section. GB is National Specialty Advisor for Perinatal Mental Health for NHS England, member of the DSMB for Brexanolone for treatment for postnatal depression, Chair for Action on Postpartum Psychosis Charity and previous Vice Chair and now elected member of the Faculty of Perinatal Psychiatry at the Royal College of Psychiatrists. GB has previously attended conferences organised by the American Centre for Psychiatry and Neurology, for which travel expenses were paid. JRT is a member of Faculty of Public Health Women and Girls Special Interest Group.
