# Supplementary material for: Prevalence and incidence of moderate and severe mental illness in the second postpartum year in England (1995–2020): a national retrospective cohort study using primary care data
Source: Lancet Reg Health Eur. 2025 May 9;53:101312. doi: 10.1016/j.lanepe.2025.101312 (PMC12136845; doi:10.1016/j.lanepe.2025.101312)
Supplement: Supplementary Table [file mmc1.docx]

## Supplementary material 1. Frequency of diagnoses, symptoms and prescriptions codes used to identify cases.

Frequencies and percentages of diagnosis, symptom, prescription codes used to identify mental illness in the second postpartum year by year of birth.

| **Year** | **Number of pregnancies** | **Diagnosis code** | **Symptom code** | **Prescription code** |
| --- | --- | --- | --- | --- |
|  |  |  |  |  |
| 1995 | 85756 | 4360 (5.1) | 1043 (1.2) | 5273 (6.1) |
| 1996 | 88742 | 4791 (5.4) | 1306 (1.5) | 6144 (6.9) |
| 1997 | 91222 | 4765 (5.2) | 1402 (1.5) | 6643 (7.3) |
| 1998 | 93913 | 5043 (5.4) | 1692 (1.8) | 7511 (8.0) |
| 1999 | 94011 | 5449 (5.8) | 1910 (2.0) | 8542 (9.1) |
| 2000 | 94949 | 5995 (6.3) | 2300 (2.4) | 9815 (10.3) |
| 2001 | 96647 | 6224 (6.4) | 2787 (2.9) | 10784 (11.2) |
| 2002 | 97954 | 6424 (6.6) | 3423 (3.5) | 11795 (12.0) |
| 2003 | 103549 | 6511 (6.3) | 4040 (3.9) | 12641 (12.2) |
| 2004 | 106097 | 6464 (6.1) | 4532 (4.3) | 13058 (12.3) |
| 2005 | 104696 | 6532 (6.2) | 4654 (4.4) | 13392 (12.8) |
| 2006 | 106854 | 6451 (6.0) | 4769 (4.5) | 14058 (13.2) |
| 2007 | 107338 | 6453 (6.0) | 4970 (4.6) | 14497 (13.5) |
| 2008 | 106804 | 6609 (6.2) | 5245 (4.9) | 15110 (14.1) |
| 2009 | 102699 | 6313 (6.1) | 5280 (5.1) | 15228 (14.8) |
| 2010 | 100356 | 5802 (5.8) | 5103 (5.1) | 14944 (14.9) |
| 2011 | 95317 | 5462 (5.7) | 4933 (5.2) | 14852 (15.6) |
| 2012 | 90235 | 5086 (5.6) | 4513 (5.0) | 13841 (15.3) |
| 2013 | 79003 | 4217 (5.3) | 3933 (5.0) | 12092 (15.3) |
| 2014 | 69505 | 3815 (5.5) | 3201 (4.6) | 10703 (15.4) |
| 2015 | 58164 | 3125 (5.4) | 2848 (4.9) | 9231 (15.9) |
| 2016 | 47385 | 2481 (5.2) | 2521 (5.3) | 8283 (17.5) |
| 2017 | 39861 | 2303 (5.8) | 2338 (5.9) | 7758 (19.5) |
| 2018 | 34098 | 1669 (4.9) | 1746 (5.1) | 6690 (19.6) |
| 2019 | 28417 | . | . | . |
| 2020 | 9182 | . | . | . |
| Total | 2132754 | 122330 (5.8) | 80470 (3.8) | 262806 (12.5) |
